# Anti-Angiogenic and Anti-Proliferative Graphene Oxide Nanosheets for Tumor Cell Therapy

**DOI:** 10.3390/ijms21155571

**Published:** 2020-08-04

**Authors:** Valeria Verde, Anna Longo, Lorena Maria Cucci, Vanessa Sanfilippo, Antonio Magrì, Cristina Satriano, Carmelina Daniela Anfuso, Gabriella Lupo, Diego La Mendola

**Affiliations:** 1 Nano Hybrid BioInterfaces Lab (NHBIL), Department of Chemical Sciences, University of Catania, Viale Andrea Doria 6, 95125 Catania Italy; verdevaleria@virgilio.it (V.V.); lorena.cucci@unict.it (L.M.C.); sanfilippo.vanessa@studium.unict.it (V.S.); 2 Department of Biomedical and Biotechnological Sciences, University of Catania, Via Santa Sofia 97, 95123 Catania, Italy; longo.anna@hotmail.it (A.L.); daniela.anfuso@unict.it (C.D.A.); 3 Institute of Crystallography—National Council of Research, via Paolo Gaifami 18, 95126 Catania, Italy; leotony@unict.it; 4 Department of Pharmacy, University of Pisa, via Bonanno Pisano 6, 56126 Pisa, Italy; lamendola@farm.unipi.it

**Keywords:** theranostics, nanomaterials, nanomedicine, HPRG, peptides, cell migration, prostaglandins, human prostate cancer cells, ROS, confocal microscopy

## Abstract

Graphene oxide (GO) is a bidimensional novel material that exhibits high biocompatibility and angiogenic properties, mostly related to the intracellular formation of reactive oxygen species (ROS). In this work, we set up an experimental methodology for the fabrication of GO@peptide hybrids by the immobilization, via irreversible physical adsorption, of the Ac-(GHHPH)_4_-NH_2_ peptide sequence, known to mimic the anti-angiogenic domain of the histidine-proline-rich glycoprotein (HPRG). The anti-proliferative capability of the graphene-peptide hybrids were tested in vitro by viability assays on prostate cancer cells (PC-3 line), human neuroblastoma (SH-SY5Y), and human retinal endothelial cells (primary HREC). The anti-angiogenic response of the two cellular models of angiogenesis, namely endothelial and prostate cancer cells, was scrutinized by prostaglandin E2 (PGE_2_) release and wound scratch assays, to correlate the activation of inflammatory response upon the cell treatments with the GO@peptide nanocomposites to the cell migration processes. Results showed that the GO@peptide nanoassemblies not only effectively induced toxicity in the prostate cancer cells, but also strongly blocked the cell migration and inhibited the prostaglandin-mediated inflammatory process both in PC-3 and in HRECs. Moreover, the cytotoxic mechanism and the internalization efficiency of the theranostic nanoplatforms, investigated by mitochondrial ROS production analyses and confocal microscopy imaging, unraveled a dose-dependent manifold mechanism of action performed by the hybrid nanoassemblies against the PC-3 cells, with the detection of the GO-characteristic cell wrapping and mitochondrial perturbation. The obtained results pointed out to the very promising potential of the synthetized graphene-based hybrids for cancer therapy.

## 1. Introduction

Graphene is a 2-dimensional (2D) carbon nanomaterial consisting of planar sheets of sp^2^-hybridized carbon atoms arranged in a honeycomb lattice [1] and due to this monoatomic layer structure is the thinnest material produced so far [2]. Over the past 15 years, graphene has attracted the attention of the whole scientific community thanks to its unique mechanical and electrochemical properties, which include high density, chemical inertness, optical transmittance, very high hydrophobicity and molecular barrier abilities [3,4,5]. It is one of the most resistant materials ever tested with tensile strengths greater than 100 GPa and a traction module of 1 TPa [6]. Other remarkable features include the high planar surface (calculated value, 2630 m^2^/g [7], which allows for its higher drug loading capacity than other nanomaterials [8] and the thermal conductivity (5000 W/mK) [9]. Such properties make graphene particularly promising for applications in several fields, like the aerospace, electronics, energy, mechanical, environmental, and food industries as well as in biomedicine [10]. However, the poor solubility and agglomeration of the nanosheets in solution, caused by van der Waals forces and π–π stacking interactions, greatly limits its uses, makes it difficult to produce and significantly impacts its toxicity [11,12]. The oxygen-functionalized graphene derivative, namely graphene oxide (GO), maintaining a similar hexagonal carbon structure to graphene, overcomes these limits [13]. GO, indeed, shows a high density of functional oxygen groups namely carboxyl (–COOH) and hydroxyl (C–OH) groups, typically located at the edges of the sheets, and carbonyl (C=O) and epoxy groups (C–O–C) on the basal plane of the graphene sheets [14].

The unique chemical and physical properties of GO and its reduced derivative (rGO) have aroused a strong interest especially for biological studies [15], since the presence of defective oxygen-bound sp^3^ carbon atoms induces a strong hydrophilicity and contributes to the formation of dispersions highly stable colloidal in aqueous solvents, preventing the uncontrolled aggregation of the nanosheets caused by van der Waals and the hydrophobic interactions [16]. Moreover, the presence of hydrophilic functional groups on the GO surface offers high versatility for the derivatization of nanosheets and makes GO a suitable platform for the development of drug delivery systems [17] with potential application in tissue engineering [17], biosensing [18], and bioimaging [19], thus opening new horizons in the field of nanomedicine. In particular, graphene oxide based targeted drug carriers are becoming of great interest in the treatment of cancer diseases [20]. To this respect biological therapies, based on the delivery of biomolecules and gene therapy as well as phototherapies against cancer, including photodynamic and photothermal treatments, which use GO as a carrier, have showed high biocompatibility and good results both in *in vitro* and *in vivo* studies [21]. Thus, Liu et al. formulated transferrin modified graphene oxide for glioma-targeted drug delivery [22], Li et al. used functionalized nano-graphene oxide particles for targeted fluorescence imaging and photothermic therapy of glioma U251 cells [23], while Song and colleagues evaluated hyaluronic acid-decorated graphene oxide nanohybrids as carriers for targeted and pH-responsive anticancer therapy [24]. Furthermore, GO shows intrinsic biological properties, including antimicrobial activity [25] and the capability to control the function of immune cells [26] and to modulate angiogenesis. This latter feature provides additional advantages in cancer therapy, since formation of new blood vessels is involved in both tumor growth and development of metastases [21,27]. Hence, the anti-angiogenic action of GO can be very effective to fight cancer. To note, there are plenty of examples in the literature on the development of GO and modified GO platforms for anti-cancer therapy [28,29,30,31].

It has been demonstrated that GO sheets present pro-angiogenic properties at low doses (1–50 ng/mL), due to the controlled production of intracellular reactive oxygen species (ROS) (H_2_O_2_ and O_2_^•−^) induced by this material, while show anti-angiogenic features at high doses (≥100 ng/mL), attributed to the excessive generation of intracellular ROS [32]. In general, the mechanisms underlying GO toxicity in addition to oxidative stress and excessive ROS production also include DNA damage, apoptosis, autophagy, and immune responses, which widely varied in relation to the physical-chemical properties of GO, such as surface chemistry, layer number, lateral dimension, and purity [33].

The histidine-proline-rich glycoprotein (HPRG) is a single polypeptide chain protein of 70–75 kDa, with a multidomain structure. In humans, the protein is synthesized in the liver and is present in plasma at relatively high concentrations of 100–150 µg/mL (1.5 µM) [34,35]. HPRG ability to simultaneously interact with several ligands suggests that it may act as an adapter molecule which regulates numerous biological processes, including blood coagulation and fibrinolysis, adhesion, and cell migration, as well as anti-/pro-angiogenic activity [36].

Indeed, the HPRG protein promotes angiogenesis by inhibiting the activity of the antiangiogenic thrombospondin-1 (TPS-1) [37], by binding to plasminogen/plasmin onto the surface of endothelial cells as well as by promoting cell migration and invasion [38], which are critical phases of the new blood vessels formation. On the other hand, HPRG has also a demonstrated antiangiogenic activity, mainly localized in its histidine-proline-rich domain (H/P) and occurring by the blocking of the interaction between fibroblast growth factor (FGF-1 and FGF-2) and heparan sulphate in the extracellular matrix (ECM) and the surface of endothelial cells [39].

The Ac–(GHHPH)_4_–NH_2_ peptide has been shown to be an active HPRG mimic system, and it has been demonstrated effective as antitumor agent in two syngeneic cancer models, namely Lewis lung cancer (3LL) and melanoma (B16F1) growth in mice [40,41].

Based on these premises, in this work GO was functionalized with a with the Ac–(GHHPH)_4_GK–NH_2_ peptide [41] covalently bound to a 5,6-carboxyfluorescein (Fam) moiety, hereinafter named Tetra(HPRG)-Fam. The integration of the therapeutic potential from both GO and the Tetra(HPRG) peptide and the imaging capability through the fluorescence of the dye-labelled peptide makes the hybrid graphene oxide-Tetra(HPRG)Fam system (hereinafter named GO@T) a potential theranostic platform. The physicochemical characterization was carried out by mean of spectroscopic analyses of UV-visible, fluorescence and ATR-FTIR, to scrutinize the hybrid biointerface between the nanosheets and the peptide molecules in terms of electron transfer processes and average peptide molecular structural conformation. In vitro cellular experiments were carried out on human neuroblastoma (SH-SY5Y) and prostate cancer (PC-3) cell lines, as cellular tumor models to test the anti-angiogenic potential of our platforms in anti-cancer therapy. Both cell lines are aggressively-growing tumors, associated also to uncontrolled tumor vascularization [42]. Moreover, PC-3 can form vessel-like structures through a process denoted as vascular mimicry [43]. As endothelial cellular model of angiogenesis, we tested also our nanoformulation on primary human retinal endothelial cells (HREC) [44,45].

Specifically, the nanotoxicity and the pro-or anti-proliferative activity were investigated for GO@T hybrids compared to bare GO sheets and to the fragment peptide. Also, the nanocomposites effect on mitochondrial dysfunctions were scrutinized by measuring the mitochondrial production of O_2_^•−^ as well as their effects on cellular migration and prostaglandin E2 (PGE_2_) release. Intracellular imaging using confocal laser scanning microscopy (LSM) was performed to evaluate the mechanism of interaction and internalization of hybrid systems on the studied cancer cell line.

## 2. Results

UV-visible, circular dichroism (CD), fluorescence and attenuated total reflectance Fourier transform infrared (ATR/FTIR) spectroscopies were used to characterize the hybrid graphene oxide nanosheets functionalized with the Tetra(HPRG)-Fam peptide (samples thereafter named GO@T). Two weight ratios for the peptide/GO mixtures were used for the preparation of the samples, namely, two concentrations of graphene oxide, i.e., 1 mg/mL (GO_A) or 0.5 mg/mL (GO_B) incubated for 2 h under stirring with a fixed concentration of peptide (0.2 mM).

**Figure 1:**
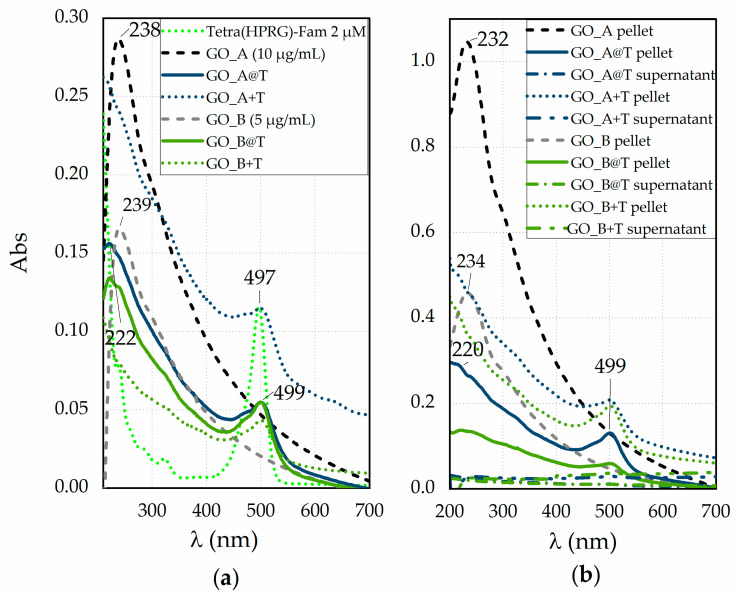
(**a**) UV-visible spectra in phosphate buffer saline solution (PBS, pH = 7.4) of GO_A and GO_B 100× diluted dispersions, both before (dashed lines for GO_A, in black, and GO_B, in grey, respectively) and after 2 h of incubation with the peptide (solid line for GO_A@T, in dark green, and GO_B@T, in light green, respectively). The reference spectra of Tetra(HPRG)-Fam (green, 100× diluted solution of that used for the incubation of GO_A and GO_B samples) and the mixtures immediately the mixing (GO_A+T, in dark green, and GO_B+T, in light green, respectively) are shown for comparison in dot line. (**b**) UV-visible spectra in PBS of the pellets for hybrid GO_A@T and GO_B@T samples (solid lines), the GO_A+T and GO_B+T mixtures (dot lines) and, for comparison, of bare GO_A and GO_B pellets (dashed lines) after two centrifugation (2700 RCF, 2 min, RT) and washing steps. The spectra of supernatants (dashed dot lines) of GO@T samples collected after the first washing step are included.

Figure 1 displays the absorption spectra of reference samples of the GO nanosheets and of the Tetra(HPRG)-Fam peptide, both before and after the mixing (Figure 1a) as well as for the corresponding pellets and supernatants recovered after the centrifugation and washing step (Figure 1b), to remove the loosely bound peptide molecules from the GO platforms.

Figure 1a shows, for the Tetra(HPRG)-Fam sample, the characteristic *π* → π* transition absorption band of the peptide bond in the 180–230 nm range as well as the absorption of the aromatic side-chains of His residues, in the range of 230–300 nm [46]. Moreover, related to the chromophore activity of the Fam moiety [47,48], two weak bands, respectively at 280 nm and in the 350–440 nm region, and a main absorption peak at 497 nm are evident. As to the GO nanosheets, the UV-visible spectra validate the approximation of using the Lambert-Beer law of solutions for these colloidal dispersions. Indeed, both GO_A and GO_B samples exhibit the same spectral features just scaled in a 1 to 0.5 ratio; namely, one absorption peak at 238 nm and a shoulder at around 300 nm, due to the π → π* and n → π* transitions, respectively [49]. The spectra of the hybrid GO@T systems display both hypochromic and bathochromic/hypsochromic shifts compared to the reference GO and Tetra(HPRG)-Fam systems, respectively. Such findings point to the occurrence of electron transfer processes at the interface established between the GO nanosheets and the dye covalently linked to the peptide. To note, the spectra of the mixture systems are mostly given by the sum of the spectra of each component, the GO and the Tetra(HPRG)-Fam, with the GO absorption bands buried by the background signal of the Fam moiety in the UV region. Also, only for GO_B+T the Fam absorption peak being significantly decreased, due to the quenching effect of GO, but with no evidence of shift in the wavelength at the maximum of absorption. These findings point to a lower level of interaction between the GO sheets and the peptide molecules in the mixture than in the hybrid nanocomposite.

After the centrifugation and washing steps (Figure 1b), the spectra of bare GO demonstrate a different relative ratio of sp^3^ (oxidized defective) to sp^2^ (graphitic-like) carbon than the pristine GO, as disclosed both by the hypsochromic shift (~6 nm) of the π → π* peak and by the slight hyperchromic shift in the shoulder related to the n → π* transitions, respectively. In particular, the heavier (and larger) GO nanosheets, which accumulate preferentially in the pellet, exhibit a relative ratio of the sp^2^ to sp^3^ domains higher than the lighter (and smaller) nanosheets that mostly gathered into the supernatant, and hence have more defective oxygen-containing groups, especially at the edges [50,51]. To note, an additional contribution to these shifts can derive by the decrease of conjugative effect of chromophore aggregation by washing-induced loss of weakly bound oxygen-containing species, which influences the π–π* plasmon peak [52]. Significantly, the pellets of GO_A@T and GO_B@T samples (Figure 1b), which display spectral features of both GO and Tetra(HPRG)-Fam components, do not show significant changes in the peak wavelength position with respect to those of the hybrid systems before the centrifugation and rinsing steps (Figure 1a). These findings point to the successful and irreversible immobilization of the peptide molecules at the GO nanosheets surface.

As to the mixtures, also in this case the spectra evidence an irreversible binding of the Fam-labeled peptide to the GO substrates. For both GO@T hybrids and GO+T mixture samples, the spectra of supernatants collected after first washing step do not display any significant indication of the presence of peptide neither of GO. By using the molar extinction coefficients calculated for Tetra(HPRG)-Fam in the hybrid nanocomposite, a peptide loading of about 95 µM for 1 mg/mL of GO in GO_A@T or GO_A+T and of 88 µM for 1 mg/mL of GO in GO_B@T or GO_B+T could be estimated, respectively.

**Figure 2:**
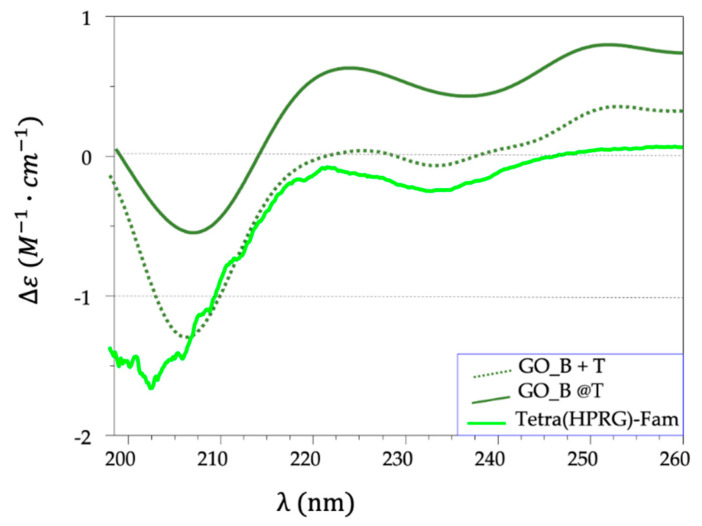
CD spectra in phosphate buffer saline solution (PBS, pH = 7.4) of Tetra(HPRG)-Fam (green, solid line, peptide concentration = 1 × 10^−5^ M) and the pellets (10× dilution) for hybrid GO_B@T (dark green, solid line) and mixture GO_B+T (dark green, dotted line) samples.

The analysis of CD spectra for both hybrids and mixture samples, in the comparison with the spectrum of the peptide alone, evidenced: (i) no detectable peptide signals for GO_A@T and GO_A+T (data not shown), likely due to the low relative concentration of the peptide compared to the GO matrix; (ii) a progressive intensity decrease and a red-shift of the minimum band at 202 nm for both GO_B+T (at 206 nm) and GO_B@T (at 207 nm) (Figure 2). The redshift is explained as result of the increase in the polyproline II (PPII) conformers [53]; the intensity change is due to the optical coupling between the peptide chromophore moieties and the GO substrates. Over all, these findings point to a strong interaction between the nanosheets and the Tetra(HPRG)-Fam, which affects at different levels (higher in the hybrids than in the mixtures) the peptide conformational structure and its freedom degrees.

**Figure 3:**
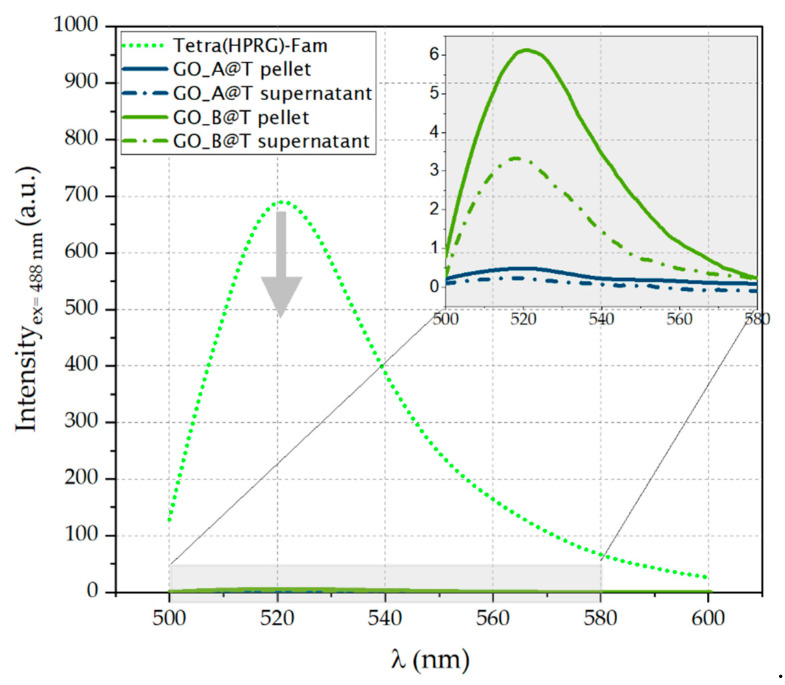
Fluorescence spectra (λ excitation = 488 nm) in PBS (pH = 7.4) of free Tetra(HPRG)-Fam peptide (dot green line) and GO@T hybrids (GO_A@T: dark green line; GO_A@T: light green line). In the inset the magnified region for the pellets (solid lines) and supernatants (dashed-dot lines).

The electron transfer processes at the interface between the GO nanosheets and the Fam-labelled peptide were confirmed by the fluorescence spectra (Figure 3), which display a strong quenching of the Fam peak at 521 nm. In particular, the fluorescein emission decreases of 99.9% in GO_A@T and to 99.1% in GO_B@T samples (see inset Figure 3), respectively.

**Figure 4:**
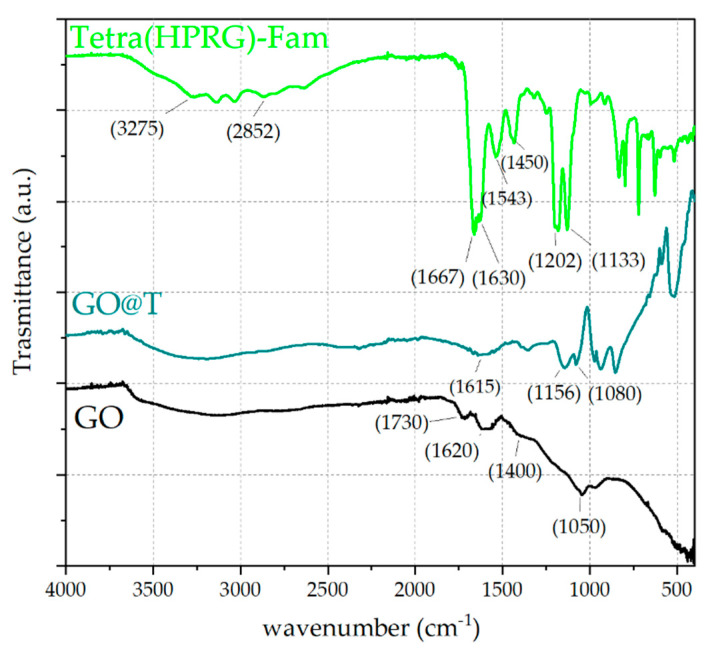
Attenuated total reflectance Fourier transform infrared (ATR/FTIR) spectra of graphene oxide (GO) (black curve), Tetra(histidine-proline-rich glycoprotein (HPRG))-Fam (green curve) and GO@T (dark cyan curve) hybrid samples.

The characterization by ATR-FTIR analyses (Figure 4) confirmed the effective immobilization of the peptide on the GO nanosheets in the hybrid nanocomposite. As to the uncoated GO, the spectrum displays a broad band in the wavenumber range of 3700–3000 cm^−1^, assigned to O-H stretching vibrations, as well as two peaks at ~1730 cm^−1^ and 1620 cm^−1^, due to the C=O stretching of the carboxylic acid groups and the aromatic C–C stretching, respectively [54]. Moreover, a weak signal at ~1400 cm^−1^ corresponding to the O–H deformation and an absorption peak at ~1050 cm^−1^ attributed to the alkoxy C–O stretching vibrations are also visible [55,56]. The spectrum of Tetra(HPRG)-Fam shows characteristic peaks from both the 5,6-carboxyfluorescein unit and the peptide backbone. Specifically, the O–H stretching vibrations in the broad band from 3600 to 3300 cm^−1^ as well as the signals at 1667 cm^−1^ and 2852 cm^−1^, which correspond respectively to the stretching of the C=O of carboxyl groups and the C–H stretching [57] are visible, together with the typical IR bands for amino acids and peptide (the “bio-fingerprint region”) at 1630 cm^−1^ (amide I) and 1543 cm^−1^ (amide II) are visible [58]. Moreover, the signals at 1202 cm^−1^ can be assigned to the C=C stretching and the N–H bending vibrations of the histidine residue, while the weak band in range of 1400 and 1465 cm^−1^ can be attributed to the C–N stretching of the proline and the sharp peak at 1133 cm^−1^ corresponds to the C–O stretching vibrations of the peptide [59].

As to the GO@T hybrids, the signals at 1156 and 1080 cm^−1^, related respectively to the C=C stretching and the N–H bending vibrations of the histidine residue confirm the presence of peptide-related bands in the nanocomposite sample. Such signals are shifted to higher wavenumbers and the peak are broader with respect to those observed for free peptide, thus suggesting the formation of hydrogen binding between the hydrophilic groups of graphene oxide and the side chain imidazole groups of histidine in the peptide [60]. Moreover, the fingerprint signals from the amide I and amide II vibrations are strongly attenuated, likely due to a different conformational state of the peptide immobilized in the adlayer on the GO surface compared to the unbound peptide molecules.

**Figure 5:**
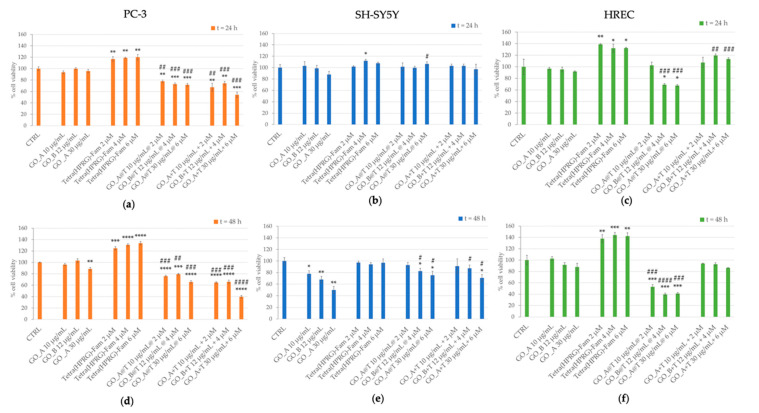
Cell viability from MTT assays of prostate cancer (**a**,**d**), neuroblastoma (**b**,**e**) and endothelial (**c**,**f**) cells incubated for 24 h (**a**–**c**) or 48 h (**d**–**f**) with the hybrid GO@T samples at increasing content of GO nanosheets (10–30 µg/mL concentration range) and Tetra(HPRG)-Fam peptide (3–6 µM concentration range). The negative control of untreated cells (CTRL) and the positive controls of cells treated with the peptide alone or the bare GO or the peptide mixtures are included as reference. Values (means ± SEM) are from three independent experiments. Results are expressed as percentage with respect to CTRL. Student’s *t*-test was used to compare cell viability measurements in all experimental conditions. (*) *p* < 0.05, (**) *p* < 0.01, (***), *p* < 0.001, (****) *p* < 0.0001 vs. CTRL, (^#^) *p* < 0.05, (^##^) *p* < 0.01, (^###^) *p* < 0.001, (^####^) *p* < 0.0001 vs. the corresponding GO reference sample.

The response of prostate cancer (PC-3), neuroblastoma (SH-SY5Y) and non-tumor endothelial (HREC) cells to the GO@T hybrid nanocomposites was investigated in terms of toxicity through MTT assay. Cell viability assays were performed on cells incubated for 24 h and 48 h with increasing concentrations of GO nanosheets (10, 12, 30 µg/mL), Tetra(HPRG)-Fam (2, 4, 6 µM) and three hybrid (GO@T) and mixture (GO+T) samples, spanning the same concentration range in the GO and the peptide (Figure 5).

In general, the cellular treatments with GO samples resulted not toxic after 24 h of incubation time for all the three cell lines tested. After 48 h of incubation, we detected a statistically significant decrease in cell viability in PC-3 (30 µg/mL GO: 88.5 ± 2.6% of CTRL; ** *p* < 0.01) and SH-SY5Y (10 µg/mL GO: 78.0 ± 5.1% of CTRL; * *p* < 0.05; 12 µg/mL GO: 68.1 ± 5.6% of CTRL; ** *p* < 0.01; 30 µg/mL GO: 50.1 ± 5.9% of CTRL; * *p* < 0.01;) but not in HRECs. Also, our findings are consistent with many other studies, reporting that GO is non-toxic for multiple cell types [61,62,63].

Interestingly, the treatment with Tetra(HPRG)-Fam alone induced an increased viability both in prostate cancer (Figure 5a, 24 h: ~120% of CTRL: ** *p* < 0.01; Figure 5d, 48 h: up to ~130% of CTRL: *** *p* < 0.001) and endothelial cells (Figure 5c, 24 h: ~130% of CTRL: * *p* < 0.05; Figure 5f, 48 h: ~140% of CTRL: ** *p* < 0.01) cells, while no significant changes in cell viability were detected in neuroblastoma cells for both 24 h (Figure 5b) and 48 h (Figure 5d) of incubation.

A comparable anti-proliferative effect was induced in PC-3 by the treatment either with the GO@T hybrids or the GO+T mixtures, for both the incubation times of 24 and 48 h. In particular, a statistically significant dose-dependent decrease of viability was detected with respect to both the untreated cells as well as the GO-treated cells, with a maximum of cytotoxicity found for the mixture of 30 µg/mL GO with 6 µM Tetra(HPRG)-Fam (24 h: 54.3 ± 4.8% of CTRL: *** *p* < 0.001; 48 h: 39.8 ± 2.2% of CTRL: **** *p* < 0.0001).

As to SH-SY5Y cells, a significant and comparable decrease in cell viability was detected only in the cells incubated for 48 h with the highest concentrations of GO@T hybrid (~80% of CTRL: * *p* < 0.05) or GO+T mixture (~88–70% of CTRL: * *p* < 0.05), respectively. To note, the same treatment conditions resulted however proliferative in comparison to the incubations with corresponding positive controls of GO.

The HREC treatments with the highest concentrations of GO@T hybrids resulted anti-proliferative both after 24 h (~70% of CTRL: * *p* < 0.05) and 48 h (~40% of CTRL: * *p* < 0.05) of incubation. On the other hand, the endothelial cells incubation with the GO+T mixture at the highest concentrations showed only a slightly proliferative effect at 24 h with respect to the positive control of GO (~120% of CTRL: ^##^ *p* < 0.01).

According to the results of anti-proliferative effects induced by GO@T hybrids in PC-3 and HREC but not in SH-SY5Y, the following investigations on the inhibition of cell migration and PGE_2_ release were carried out only on the prostate cancer and endothelial cells.

**Figure 6:**
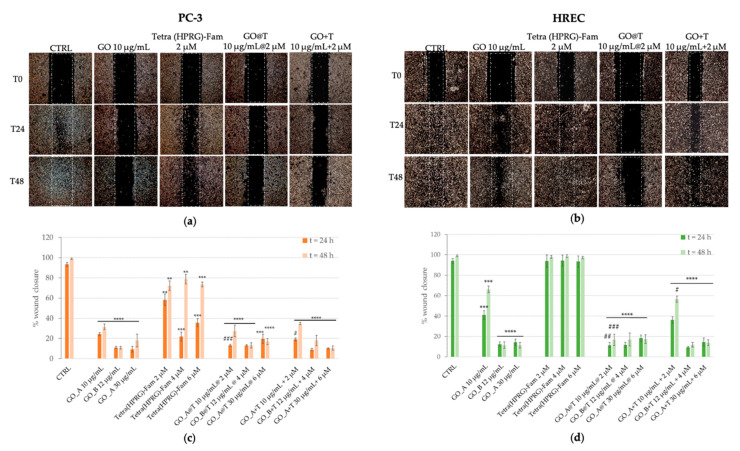
Representative micrographs (**a**,**b**) and quantitative analysis of cell migration (**c**,**d**) for prostate cancer (**a**,**c**) and endothelial (**b**,**d**) cells in absence (negative control, CTRL) or in presence of the different treatments at time 0, 24 h and 48 h after scratch: GO nanosheets (10, 12, 30 µg/mL), Tetra(HPRG)-Fam peptide (2, 4, 6 µM), GO@T hybrids (10 µg/mL 2 µM, 12 µg/mL 4 µM, 30 µg/mL 6 µM of GOpeptide concentration) and GO+T mixtures. PC-3 and HREC cells were wounded as described in the Materials and Methods section. The quantitative analysis of migration assay (wound edge advancement in percent vs. time) is expressed as means values (± SEM) from three independent experiments. Statistical analysis was performed by pairwise Student’s *T*-test results are expressed as percentage of wound closure with respect to time 0. (**) *p* < 0.01, (***) *p* < 0.001, (****) *p* < 0.0001 vs. CTRL, (^#^) *p* < 0.05, (^##^) *p* < 0.01, (^###^) *p* < 0.001 vs. the corresponding GO reference sample.

The modulation of PC-3 and HREC scratch crossing was monitored for 24 h and 48 h after monolayer wounding (Figure 6).

Representative photographs and the quantitative analysis of wound healing for PC-3 (Figure 6a,c) and HREC (Figure 6b,d) incubated in absence (negative control) or in presence of the positive controls of GO nanosheets (10, 12, 30 µg/mL),Tetra(HPRG)-Fam (2, 4, 6 µM) and the corresponding GO+T mixtures are shown in the comparison with those of cells incubated with the hybrid GO@T samples.

The control untreated PC-3 cells were able to cross the wound at 24 h, until the wound closed after 48 h. Conversely, the migration ability of PC-3 treated with GO was reduced significantly in comparison with control cells. A dose-dependent partial inhibition of cell invasion was observed in presence of Tetra(HPRG)-Fam, especially evident at 24 h of incubation (2 µM: ~70% of CTRL: ** *p* < 0.01; 4 and 6 µM: ~30% of CTRL: *** *p* < 0.001) than after 48 h of treatment (2, 4, and 6 µM: ~80% of CTRL: ** *p* < 0.01). The treatment of PC-3 either with the GO@T hybrids or the GO+T mixtures induced a significant and comparable reduction of the wound closure (at the two highest concentration tested: less than 20% of CTRL: **** *p* < 0.0001), indicating that the association of the peptide to the GO nanosheets efficiently reduces the PC-3 cells migration, inhibiting their metastatic potential.

HREC migration was modulated differently from cancer cells. Indeed, whereas the cells incubation with GO@T hybrids exhibited a significant inhibitory effect on wound closure for all three concentration tested (less than 20% of CTRL: **** *p* < 0.0001), and no differences in comparison with the negative control of untreated cells were observed for the cells incubated with the peptide alone, both the GO and the GO+T mixtures showed a similar trend, i.e., a partial inhibition for the lowest concentration (10 µg/mL: ~40% at 24 h and ~60% at 48 h of CTRL: *** *p* < 0.0001) and a very efficient inhibition of wound closure (less than 20% of CTRL: **** *p* < 0.0001) at the other two concentrations for both incubation times.

Inflammation is increasingly recognized as a critical mediator of angiogenesis [64]. It has been demonstrated that PGE_2_ promotes the in vitro tube formation of human microvascular endothelial cells via activation of EP4 receptors and by triggering PKA Cγ signaling pathway, *ex vivo* vessel outgrowth of aortic rings, and *in vivo* angiogenesis [65,66,67]. Moreover, during the inflammatory process, immune cells secrete pro-angiogenic factors for the promotion of new vessel formation [64].

The ability of GO@T hybrids to modulate the release of PGE_2_ in PC-3 and HREC was investigated as marker for the activation of inflammatory processes, which can have a crucial role both in carcinogenesis and cancer progression as well as in the angiogenic switch-on mechanism.

Cell culture supernatants collected after incubation of PC-3 or HREC cells for 48 h in absence (control) or in presence of GO nanosheets, Tetra(HPRG)-Fam, the hybrid GO@T or the mixture GO+T, were assayed for their PGE_2_ levels (Table 1 and Table 2).

As to prostate cancer cells, upon the incubations with the different samples, the PGE_2_ production by PC-3 cells decreased in comparison to untreated control cells. Specifically, Table 1 shows a dose-dependent effect (about 35–40%) for the treatments with GO, and a reduction in the PEG_2_ release by about 26% for Tetra(HPRG)-Fam, and by about 39% for the GO+T mixture, respectively. Finally, the treatments with GO@T hybrids determined a significant decrease by about 60% for all used concentrations.

Analogous investigation on HREC (Table 2) showed that PGE_2_ levels were significantly reduced after the cellular treatments with GO or GO+T (by 20–29% or 39%, respectively) but not after incubation with the three different concentrations of Tetra(HPRG)-Fam. On the other hand, a significant and large decrease in comparison to untreated cells (by 64%), comparable to that found in the PC-3 case, was detected for HREC cells incubated with GO@T hybrids.

These results confirmed the role played in prostate cancer by PGE_2_, the most abundant pro-inflammatory mediator, promoting cancer cell invasion by induction of protein and mRNA expression of metalloproteinases, and, in turn, allow for the proliferation and migration of PC-3 cells [68]. Moreover, the nanocomposites of GO and Tetra(HPRG)-Fam peptide, although to different extend, were able to modulate the PGE_2_ release and thus the new vessel formation as well as the tumor progression. Interestingly, the same trend measured in HREC for both the cell migration and the PEG_2_ release upon the treatment with the peptide alone, i.e., no significant changes with respect to the negative control of untreated cells, points to a certain role of the switch-on process of angiogenesis in the mechanism involved.

The ability of GO@T to modulate the release of PGE_2_ in PC3 cell makes them potential anti-inflammatory agents for use in the conditions under which inflammation amplifies the pathological process. In particular, chronic inflammation is a risk factor for the development and progression of prostate cancer [69]. It has been demonstrated in PC3 that PGE_2_ binds to a prostaglandin transporter which thus determines an increase in their intracellular concentration and, with an intracrine mechanism, they induce the proliferation, migration and invasion of PC-3 tumor cells [70].

**Figure 7:**
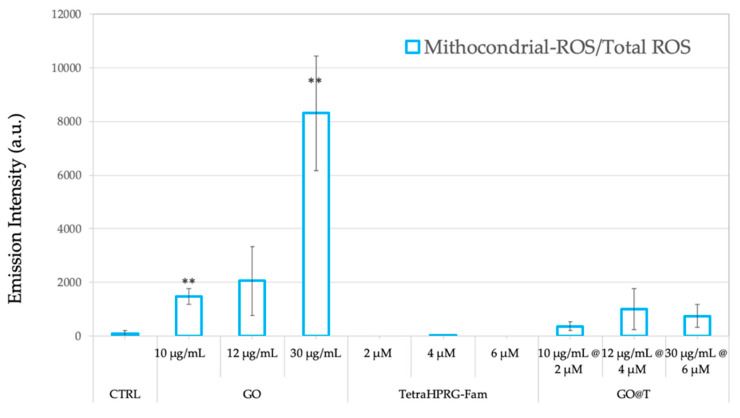
Ratio of mitochondrial-reactive oxygen species (ROS)/total-ROS levels measured on prostate cancer cells (PC-3) measured by the MitoSOX and dichlorohydrofluorescein (DCF) fluorescence assays. Cells were incubated for 24 h in absence (negative control, CTRL) or in presence of the different treatments: GO nanosheets (10, 12, 30 µg/mL), Tetra(HPRG)-Fam peptide (2, 4, 6 µM) and hybrid GO@T samples (10 µg/mL-2 µM, 12 µg/mL-4 µM, 30 µg/mL-6 µM of GO-peptide concentration). Values (means ± SEM) are from three independent experiments. Statistical analysis was performed by One-way ANOVA test. Symbols indicate the significance versus CTRL: ** *p* < 0.01. Results are expressed as ratio of MitoSOX with respect to DCF emission intensities.

To shed light on the molecular mechanisms involved in toxicity induced by the hybrid GO@T platforms, the oxidative stress induced by the production of reactive oxygen species (ROS) was measured through the fluorescent assays of MitoSOX Red, and dichlorohydrofluorescein (DCF), to measure mitochondrial superoxide, and cytosol hydrogen peroxide production, respectively. Hence, PC-3 cells were treated for 24 h with three different concentrations of GO, Tetra(HPRG)-Fam and GO@Tetra(HPRG)-Fam. Figure 7 shows that the PC-3 cells treatment with GO nanosheets increases the ROS in a dose-depended manner, in agreement with the literature on the GO-induced cytotoxicity mechanism by intracellular ROS production [27,49]. On the other hand, no significant ROS production was detected upon the cells treatment by Tetra(HPRG)-Fam as well as by the hybrid GO@T samples in the concentration range tested.

**Figure 8:**
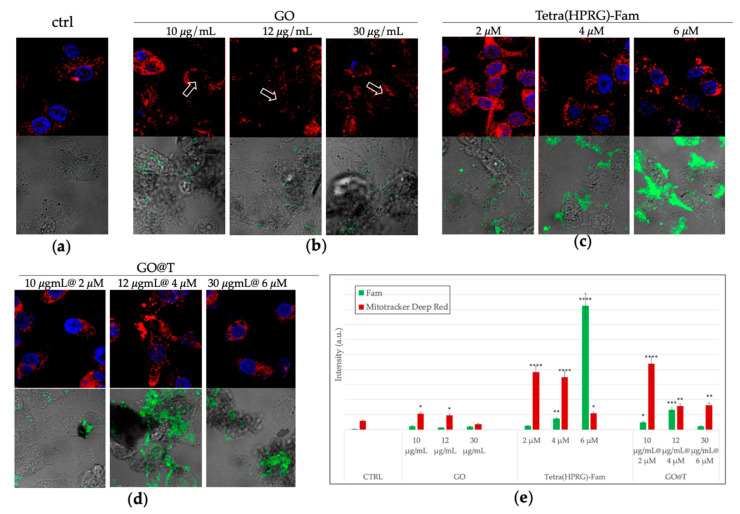
Laser scanning microscopy (LSM) micrographs of PC-3 cells incubated for 24 h in absence (**a**: CTRL) or in presence of the different treatments: GO nanosheets (**b**: 10, 12, 30 µg/mL), Tetra(HPRG)-Fam peptide (**c**: 2, 4, 6 µM) and hybrid GO@T samples (**d**: 10 µg/mL-2 µM, 12 µg/mL-4 µM, 30 µg/mL-6 µM of GO-peptide concentration). For each treatment condition, the merged confocal images of nuclei (Hoechst, λex/em = 405/425–450 nm) and mitochondria (MitoTracker Deep Red, λex/em = 633/650–700) (upper panel), and the merged fluorescence image of the dye-labelled peptide (Fam, λex/em = 543/550–600 nm) and the optical bright field micrograph (lower panel) are shown. Scale bar 30 µm. The white arrows guide the eye to the GO sheets. In (**e**): quantitative analysis of Fam and MitoTracker Deep Red emission intensity. Results are presented as mean +/-SEM from experiments in triplicate and normalized with respect to the control untreated cells. Asterisks represent the correlation significant at the (*) *p* < 0.05, (**) *p* < 0.01, (***) *p* < 0.001 (****) *p* < 0.0001 vs. CTRL, (One-way ANOVA).

To tackle with the correlation between the pro-tumor effect of PGE_2_ and the particular ability of GO@T to reduce their synthesis we carried out intracellular imaging LSM to evaluate the cellular uptake of the GO nanosheets decorated by the fluorescent Tetra(HPRG)-Fam peptide as well as to image the perturbation on the mitochondria, as analyzed by the organelle staining by the MitoTracker Deep Red fluorescent probe. Figure 8 shows the representative LSM micrographs of PC-3 cells after 24 h of treatment followed by cellular staining and fixation.

As to the GO-treated cells (Figure 8b), several dark areas in the optical light field micrographs (not observed in the control untreated cells, Figure 8a), show the cellular wrapping by the GO sheets aggregates, which is one of the mechanism invoked for the GO cytoxicity [71]. Interestingly, such GO sheets are detected in close contact to the cell membrane but also intracellularly, as evidenced by the shadowing of the nuclear staining (see arrows in Figure 8a), thus pointing to point to the effective cellular uptake of the GO nanosheets. As to the cells incubated with the free peptide (Figure 8c), a green fluorescence that increases in a dose-dependent manner is clearly visible in the cell cytoplasm and, mostly, accumulating at the cell membrane for the highest peptide concentration tested. Finally, the cells incubated with the three GO@T samples at the different relative concentration of peptide and GO, show a more diffuse green fluorescence inside the cells, as well as peptide-decorated (i.e., green emitting) GO aggregates that wrap/trap the cells in a ‘sheet-form blanket’. To note, the isoelectric pH value of 7.9 calculated for the free peptide at the physiological pH suggests an interaction driven by electrostatic forces with the negatively charged cell membranes. On the other hand, for the GO-immobilized peptide a more effective transport into the cells is offered by the graphene-based platform [20]. These findings validate the self-assembly-based approach used to fabricate the nanocomposite for their use in the theranostics concept.

The quantitative analysis (Figure 8e) confirms the effective internalization of the free peptide in a dose dependent manner, and suggest, by the increase of the detected emission of the MitoTracker Deep Red probe compared to the control untreated cells, a general mitochondrial insult, especially for the bare GO nanosheets and the GO@T hybrid with the lowest concentration of peptide among those tested.

## 3. Discussion

Small peptide molecules preferentially interact via electrostatic interaction with the edge or basal plane surface of the GO nanosheets. Indeed, the hydrophilic groups of graphene oxide, such as –COOH and –OH, allow for the versatile immobilization of biomolecules via either covalent grafting or non-covalent interactions that include hydrogen bonds, ion–dipole, or van der Waal forces, while the free surface π electrons are capable of forming π–π and CH–π and interactions [49,72]. Noteworthy, aromatic amino acids, such as the histidine included in the aminoacidic sequence of the Tetra(HPRG) peptide, preferably orient themselves in parallel with respect to the basal plane of the graphene sheets, through predominant π–π interactions [73,74].

A strong and irreversible interaction between the physisorbed Tetra(HPRG) peptide and GO was detected by means of UV-visible (Figure 1) and fluorescence (Figure 3) spectroscopies, as demonstrated in terms of electron transfer processes between the nanosheets and the 5,6-carboxyfluorescein fluorophore moiety covalently bound to the Tetra(HPRG) peptide sequence.

In particular, the bathochromic shift (~2 nm) and, most noticeably, the hypochromic shift of the Fam-related main absorption peak at ~500 nm, pointed to the significant decrease, in a GO-dose dependent manner, of the molar extinction coefficient from the value of ε_497_ = 5.6 × 10^5^ M^−1^cm^−1^ of the free peptide to ε_499_ = 1.3 × 10^5^ M^−1^cm^−1^ and ε_499_ = 1.7 × 10^5^ M^−1^cm^−1^ in the GO_A@T and GO_B@T hybrid samples, respectively. Moreover, while the bare GO_A and GO_B nanosheets have similar molar extinction coefficients for the GO characteristic π → π* transition peak, as calculated by using the Lambert-Beer law (ε_238_ ~3 × 10^2^ mg^−1^ mL cm^−1^), the hybrid GO@T samples exhibit both hypsochromic (~16 nm) and hypochromic shifts, the latter effect being depending on the GO concentration, with the values of ε_232_ = 73 mg^−1^ mL cm^−1^ for GO_A@T and ε_232_ = 93 mg^−1^ mL cm^−1^ for GO_B@T estimated from the spectra of the hybrid samples subtracted by that of the free peptide.

GO, which is itself fluorescent, is a quencher for the fluorescence signals of fluorophores, with a quenching efficiency superior to that of conventional organic molecules [75]. Its quenching mechanism is related either to fluorescence resonance energy transfer (FRET) [76] or photo-induced electron transfer (PET) processes [77]. Hence, when the GO nanosheet and the fluorophore molecules are close to each other, the energy or excited electron transfers from the fluorophore (donor) to the GO (acceptor) and the fluorescence signal of the fluorophore is quenched. GO is largely used as energy acceptor for the fabrication of biosensors [78]. Our findings on the strong quenching of the Fam fluorescence (<99% decrease in the emission intensity measured in the absence of GO) in the GO@T hybrids strongly support the successful and irreversible immobilization of the Tetra(HPRG)-Fam molecules on the GO nanosheets (Figure 3).

The CD (Figure 2) and ATR-FTIR (Figure 4) spectra also confirmed the successful immobilization of the peptide molecules at the GO surface, with conformational changes of the peptide molecules due to the strong interaction with the GO nanosheets. In particular, in the infrared spectra, the shifts to higher wavenumbers and the peak broadening of the signals at 1156 and 1080 cm^−1^, related respectively to the C=C stretching and the N–H bending vibrations of the histidine residue, point out to the formation of hydrogen bonds between the hydrophilic groups of graphene oxide and the side chain imidazole groups of histidine in the peptide [60]. Also, the spectral changes observed in the protein-fingerprint region of amide I and amide II stretching vibrations pointed to significant conformational changes of the peptide molecules in the adlayer at the surface of the GO compared to the unbound form of the free peptide in solution. Specifically, the amide I vibration, absorbing near 1650 cm^−1^, arises mainly from the C=O stretching with minor contributions from the out-of-phase C–N stretching. The amide II, absorbing near 1550 cm^−1^, is the combination of N–H and C–N stretching vibration with smaller contributions from the C–O, C–C and N–C stretching vibrations. Both amide I and amide II vibration peaks of proteins may provide valuable structural information and secondary structure prediction [59]. For instance, the amide I peak may encompass different conformational contributions that represent extended strands, β-turns, 3(10)-helix, polyproline I, and polyproline II [79]. The HPRG domain has been reported to have a predominant polyproline II conformation rather than random coil one for the high content of proline residues [80]. We found by CD analyses supporting evidences on the presence of PPII conformers of the free Tetra(HPRG) peptide in equilibrium with the random coil configuration. Indeed, the CD spectrum of the free peptide (Figure 2), shows a broad negative peak at 202 nm, a maximum at 222 nm, and another minimum at 234 nm. This combination has been shown to occur in polypeptides with extended chain polyproline II conformation, whereas the random conformation has the minimum is at 199 nm [81].

To note, the HPRG domain is able to bind different ligands by exploiting the imidazole side chain, partially protonated at physiological pH, and the conformational features of its secondary structure. The HPRG protein has a multidomain structure composed of 2 N-terminal regions (called N1 and N2) homologous to cystatin but lacking the function of inhibitor of cysteine proteinase; a central element, histidine rich region (HRR) flanked by 2 regions rich in proline (PRR1 and PRR2) and a C-terminal domain or (C) [82]. This domain also prevents heparanase-mediated release of angiogenic growth factors from ECM by blocking the heparanase cleavage sites in the EPM heparan sulphate [83] and by binding to tropomyosin on the cell provides antiangiogenic signals to endothelial cells [41]. Interestingly, these processes could be enhanced by high extracellular Zn^2+^ (generally provided by platelets) and low pH levels, which cause conformational changes in the molecule [34]. Noteworthy, the multidomain structure of HPRG allows for its interaction with a variety of molecules such as heme group, fibrinogen, heparinase, thrombospondin (TSP), divalent metal cations zinc, copper, mercury, cadmium and nickel, but also with associated molecules to cells, including heparan sulphate (HS), tropomyosin ATP synthase, DNA [36]. Specifically, HPRG has been detected on the surface of the cells involved in the immune response such as: Leukocytes, macrophages and monocytes, as well as in the platelet and megakaryocyte granules [35]. Numerous studies have demonstrated the effectiveness of HPRG-derived peptides in inhibiting tumor cell proliferation indirectly, by reducing angiogenesis and starving the tumor. To date, there are no studies conducted on tumor cells after incubation with the Tetra(HPRG) peptide or graphene-based nanocomposites either of the whole protein or of the peptide.

The different viability results (Figure 5) showed by the cells incubated with GO-immobilized peptide, both hybrids GO@T and mixtures GO+T, compared to the free Tetra(HPRG)-Fam peptide could be therefore related to the different binding capabilities of the HPRG backbone in the free and surface-immobilized conformational state.

Specifically, the opposite trends detected on both PC-3 and HREC, i.e., proliferative effect for the peptide alone and anti-proliferative effect for the GO-immobilized peptide, can be related to the equilibrium shift for the latter towards the PPII configuration. To note, according to the confocal imaging analyses (Figure 8), the cells exhibited a dose-dependent cellular uptake of the free peptide with preferential gathering at the cell membrane, while a more diffuse cytoplasm internalization was observed for the GO-immobilized peptides. Accordingly, different signaling pathways resulting in the proliferative or anti-proliferative effects could be activated.

The effects induced by the GO@T hybrids and GO+T mixtures on PC-3 and HREC cell viability confirm that, depending on the incubation time and the dose of treatment, it could be also used as an anti-cancer molecule and not only as a drug-delivery agent [63]. What is more, the combination Tetra(HPRG)-Fam peptide to the GO platform in the GO@T hybrids blocked the cancer cells proliferation to a greater extent than each individual component. In the other hand, in the angiogenic cellular model, the GO@T hybrids inhibit the endothelial cell proliferation while the GO+T mixture do not, thus highlighting a promising potential to block unwanted vessel formation.

The different effects of GO and GO@T hybrids on PC-3, SH-SY5Y and HREC are in agreement with other studies showing that graphene oxide inhibits tumor-sphere formation in different cancer types (breast, ovarian, prostate, lung, and pancreatic cancers, as well as glioblastoma), by inhibiting several signal transduction pathways, but with no toxic for normal fibroblasts [84]. Also, the time- and dose-dependent response on the treatment of SH-SY5Y cells with GO is in agreement with literature data [85] and previous works from this group which demonstrated that the GO toxicity in neuroblastoma cells depends on the functional groups and the oxidation level of GO [27,50]. These findings are in general very promising since they elucidate on the potential of graphene oxide as an effective non-toxic therapeutic strategy for the eradication of cancer cells.

As to the strong inhibitory effect of the migration of PC-3 and HREC cells in the presence of GO or GO@T hybrids (Figure 6), we found a consistent trend with the PEG_2_ release (see Table 1 and Table 2), with a significant inhibitory effect in both the cell types.

For PC-3, the inhibition in cell migration can be due also to the inhibition of the electron transport chain, as highlighted by the high levels of ROS produced in the cells after incubation with GO (Figure 7). This hypothesis is confirmed by other studies, reporting a blockage of the electrons transfer to the iron-sulfur centers, since GO would subtract electrons, having a higher electron affinity than the iron-sulfur centers [86]. Prolonged damage over time causes inactivation of the free radical scavenger enzymes (superoxide dismutase and glutathione peroxidase) and consequently damage to proteins, DNA and lipids, which greatly thereby influencing the cell metabolism and signaling pathways [84]. Moreover, GO exposure can induce phosphorylation of ERK signaling molecules, which are related to cell cycle regulation, triggering apoptosis [87]. The reduced production of ATP by oxidative phosphorylation would result in an impairment in the F-actin cytoskeleton assembly, which is an ATP-consuming process, thus inhibiting cancer cells migration. Interestingly, the increased ROS levels for cells incubated with the GO@T hybrids is less evident than the treatment with the GO alone. Such a result is most likely due to a protective effect of the peptide which covers the surface of GO sheets thereby reducing the interaction with the oxygen molecules and as consequences the ROS production [88]. The generation of ROS by the GO, indeed, may be mediated by the adsorption of O_2_ on its surface to form a surface-bound C(O_2_) intermediate which is capable to oxidize the glutathione enzyme GSH to GSSG, thus restoring the carbon surface to its original state and releasing H_2_O_2_ [88].

Since the presence of the peptide in GO@T hybrid led to a reduction in the release of free radicals than the treatment with the GO alone, the inhibition of the PC-3 cells migration in the presence of the hybrid could be due to a mechanism not strictly related to the reduction of the ATP generation. One of the mechanisms proposed in modulating tumor cell migration is the activation of signal pathways, involved in the inflammatory process. It has indeed been demonstrated that PGE_2_ is the most abundant pro-inflammatory mediator in prostate tissues and its levels are increasing in prostate carcinoma [89]. Moreover, it has been shown that cAMP-PKA/PI3K-Akt signaling pathway, activated in prostate cancer cells by the binding of PGE_2_ to its EP3 receptor, is involved in PC-3 cells proliferation [68]. The strong inhibition of the PGE_2_ release by PC-3 (Table 1) and HREC (Table 2) treated with GO@T, may provide novel insight into the therapeutic potential of this hybrid nanocomposite. Indeed, GO triggers profound effects through the metabolic reprogramming of the cell, thereby exerting its anti-inflammatory effects through the downregulation of specific genes, leading to activation of the transcription factor nuclear factor erythroid 2-related factor 2, which inhibited expression of pro-inflammatory cytokines such as IL-1β and IL-6 [90]. A down-regulation by GO, via epigenetic mechanisms, of cyclooxygenase 2 (cox2), releasing PGE_2_, in human embryonic kidney 293T cells has been demonstrated [91]. The ability of GO to trigger physical interactions between the downstream factors and the cox2 promoter gives GO peculiar characteristics that make it an effective molecule in the treatment of tumor forms. In the same study, aminated GO and poly (acrylic acid)-functionalized GO were demonstrated to reduce the inflammatory response, with a weaker effect on chromatin architecture. Therefore, GO-mediated chromatin interactions may minimize toxicity in practical applications.

The association between Tetra(HPRG) peptide and the ROS-producing GO nanosheets, with the related enhanced cellular uptake and mitochondrial perturbation (Figure 8) are therefore able to strongly inhibit both the tumor cells proliferation and the cell migration process. Mitochondria, in fact, are intracellular organelles involved in energy metabolism, which show a central role in the ATP synthesis. According to the data from mitochondrial organelle staining shown in the Figure 8, the GO@T hybrids at the higher concentrations of peptide and GO interfere less with mitochondrial activity than the peptide and GO alone.

## 4. Materials and Methods

### 4.1. Chemicals

Graphene oxide water dispersion (0.4 wt% concentration) was purchased from Graphenea Inc., (Gipuzkoa, Spain). TETRA-HPRG (Ac-(GHHPH)_4_G-NH_2_ with the addition of 6-Fam (6-carboxyfluorescein) were purchased from Caslo (Kongens Lyngby, Denmark). Phosphate buffered saline purchased by Sigma-Aldrich (St. Louis, MO, USA). Ultrapure Milli-Q water was used (18.2 mΩ∙cm at 25 °C, Millipore, Burlington, MA, USA). RPMI 1640, Dulbecco’s modified eagle medium (DMEM)-F12, fetal bovine serum (FBS), penicillin streptomycin solution and amphotericin solution for cell cultures. 2′,7′-dichlorofluorescein (DCF) were purchased from Sigma-Aldrich (St. Louis, MO, USA). MitoSOX™ red mitochondrial superoxide indicator and 2′-[4-ethoxyphenyl]-5-[4-methyl-1-piperazinyl]-2,5′-bi-1H-benzimidazole trihydrochloride trihydrate (Hoechst33342) were purchased from Thermo Fisher Scientific (Waltham, MA, USA).

### 4.2. Synthesis of GO@T Hybrid

The aqueous dispersion GO was dried 70 °C, 400 rpm overnight in Termomixer (Thermomixer Comfort model, Eppendorf, Hamburg, Germany) and 3 mg of GO dried were resuspended in Phosphate Buffered Saline (PBS) 10 mM to reach a final concentration of 1 mg/mL. Therefore, to obtain homogeneous dispersion, GO dispersion was sonicated in Labsonic Ultrasound bath at 59 Hz for 120 min maintaining the temperature at 25 °C. The obtained dispersion has a characteristic dark brown coloration. For the synthesis of the GO@T hybrid nanosystem, 20 µL of peptide (10 mM) in Milli-Q ultrapure water was added to 1 mL of the GO dispersion (1 mg/mL or 0.5 mg/mL, for GO_A@T or GO_B@T, respectively). The sample were incubated for 120 min in Thermomixer (Thermomixer Comfort model, Eppendorf) at 37 °C and 400 rpm. To remove the excess of reactants, the synthetized hybrid system was washed with PBS (10 mM) twice by centrifugation (2 min at 2700 RCF). The same washing procedure was also applied to the GO alone.

### 4.3. UV-Visible and Fluorescence Spectroscopy

UV-visible spectra of the samples in PBS were recorded using spectrometer Lambda S2 (Perkin Elmer, Waltham, MA, USA) and quartz cuvettes with an optical path length of 0.1 cm in the range of 200–700 nm. Fluorescence spectra were recorded on a LS55 (Perkin Elmer, Waltham, MA, USA) fluorimeter using quartz cuvettes with an optical path length of 0.1 cm.

### 4.4. ATR/FTIR and CD Spectroscopies

The vibrational spectra were recorded using a Perkin Elmer FT-IR spectrophotometer (Spectrum Two, Waltham, MA, USA) in the range of 4000–400 cm^−1^. To carry out the measures the solid samples of GO and GO@T hybrid dried in Thermomixer (Thermomixer Comfort model, Eppendorf, Hamburg, Germany) at 70 °C and 400 rpm overnight, have been placed on the surface of the crystal and then locked with a “clutch-type” lever before starting the measure. Each spectrum was acquired at a resolution of 2 cm^−1^ (10 scans).

The CD spectra of samples, both GO@T hybrids and GO+T mixtures, were obtained at 25 °C under a constant flow of nitrogen on a Jasco model 810 spectropolarimeter at a scan rate of 50 nm min^−1^ and a resolution of 0.1 nm. The path length was 1 cm. The spectra were recorded as average of 10 scans in the 190–260 nm range.

### 4.5. Cell Cultures and Maintenance

Prostate cancer cells (PC-3) and neuroblastoma (SH-SY5Y) cells were cultured in 25 cm^2^ tissue-culture treated Corning^®^ flasks (Sigma-Aldrich, St. Louis, MO, USA) in RPMI-1640 and Dulbecco’s modified eagle medium (DMEM)-F12, respectively. The medium was supplemented with 10% *v/v* fetal bovine serum (FBS), and contained 2 mM L-glutamine, 100 IU/mL penicillin and 0.1 mg/mL streptomycin. HREC cells were cultured in EGM-2 medium supplemented with 5% FBS. Cells were grown in an incubator (Heraeus Hera Cell 150C incubator), under a humidified atmosphere at 37 °C in 5% CO_2_.

### 4.6. MTT Assay

For cell viability assays, the 3-[4,5–dimethylthiazol-2-yl]-2,5-diphenyl tetrasodium bromide (MTT) assay was used (Chemicon, Temecula, CA, US). Cell lines were seeded in 96-well plates at the cell density per well of 1.5 × 10^4^ for PC-3 and HREC cells and 2 × 10^4^ for SH-SY5Y cells, respectively. Cells were incubated overnight at 37 °C before experiment. Afterwards, cells were treated for 24 h and 48 h in the absence (negative control) or the presence of GO nanosheets (10, 12, 30 µg/mL), Tetra(HPRG)-Fam peptide (2, 4, 6 µM) and hybrid GO@T samples (10 µg/Ml-2 µM, 12 µg/mL-4 µM, 30 µg/mL-6 µM) and the corresponding GO+T mixtures. After incubation periods, 10 µL of MTT reagent (5 mg/mL) were added to each well and the cells were incubated for 3 h at 37 °C. The formazan crystals were solubilized with 100 µL DMSO and plates were shaken for 10 min. The absorbance was measured at 570 nm with plate reader (Synergy 2-bioTek).

### 4.7. Cell Migration

PC-3 and HREC migration was measured using a standard scratch assay, performed as previously reported [92]. Cells were incubated for 24 h and 48 h in the absence (negative control) or the presence of GO nanosheets (10, 12, 30 µg/mL), Tetra(HPRG)-Fam peptide (2, 4, 6 µM) and hybrid GO@T samples (10 µg/m-2 µM, 12 µg/mL-4 µM, 30 µg/mL-6 µM) and the corresponding GO+T mixtures. Migration was followed by an inverted Leica DM IRB microscope equipped with CCD camera. Time zero represents the time immediately after the scratch for all conditions.

### 4.8. PGE_2_ Assay

To determine PGE_2_ release, PC-3 and HREC were incubated for 48 h in the absence (negative control) or the presence of GO nanosheets (10, 12, 30 µg/mL), Tetra(HPRG)-Fam peptide (2, 4, 6 µM) and hybrid GO@T samples (10 µg/mL-2 µM, 12 µg/mL-4 µM, 30 µg/mL-6 µM of GO-peptide concentration). Aliquots of culture medium were analyzed by using ELISA kits (Cayman Chemical, Ann Arbor, MI, USA), according to the manufacturer’s instructions. Three different experiments were analyzed in triplicate.

### 4.9. Confocal Microscopy Analysis

LSM imaging was performed with an Olympus FV1000 confocal laser scanning microscope (LSM), equipped with diode UV (405 nm, 50 mW), multiline Argon (457 nm, 488 nm, 515 nm, total 30 mW), HeNe(G) (543 nm, 1 mW) and HeNe(R) (633 nm, 1 mW) lasers. An oil immersion objective (60xO PLAPO) and spectral filtering systems were used. The detector gain was fixed at a constant value and images were collected, in sequential mode, randomly all through the area of the well. To perform the experiment PC-3 cells were seeded in glass bottom dishes (WillCo-dish^®^, Willco Wells, B.V., Amsterdam, The Netherland) with 12 mm of glass diameter at a density of 3 × 10^4^ cells per dish in RPMI-1640 medium supplemented with 1% FBS. Thereafter, cells were treated for 24 h in the absence (negative control) or the presence of GO, Tetra(HPRG)-Fam peptide, the hybrid GO@T and the GO+T mixtures. Before fixing, cells were stained with nuclear dye Hoechst33342 (0.251 µg/mL) and MitoTracker Deep Red (200 nM). Cells were rinsed with fresh PBS and cellular fixation was performed with high purity paraformaldehyde (2% *w/v*) in PBS (pH 7.3).

### 4.10. Total and Mitochondrial ROS Production

The oxidative stress induced by the cell treatment with GO, Tetra(HPRG)-Fam and the hybrid GO@T was evaluated through DCF assay and MitoSOX assay on PC-3 cells measuring the level of reactive oxygen species (ROS) produced. To perform the assay, cells were plated into a 96-well plate in complete medium at a density of 10 × 10^3^ cells per well. Cells were treated for 24 h in 3 replicate wells with: GO nanosheets (10, 12, 30 µg/mL), Tetra(HPRG)-Fam peptide (2, 4, 6 µM) and hybrid GO@T samples (10 µg/mL-2 µM, 12 µg/mL-4 µM, 30 µg/mL-6 µM of GO-peptide concentration). The incubation was carried out in complete medium supplemented with 1% FBS. Then, cells were stained with 0.12 µg/mL Hoechst33342 for 20 min, 3 µM 2′,7′-dichlorofluorescein (DCF) for 15 min (total ROS) or 5 µM MitoSOX for 5 min (mitochondrial O_2_^•−^) at room temperature and analyzed by measuring the fluorescence emission (λex = 493 nm, λem = 523 nm for the nuclear staining; λex = 493 nm, λem = 523 nm for DCF; λex = 510, λem = 580 for MitoSOX, respectively) using a fluorescence spectrophotometers a LS55 (Perkin Elmer, Waltham, MA, US) and quartz cuvettes with an optical path length of 0.1 cm. Results are normalized to the Hoechst emission and represented as the increase in DCF or MitoSOX signals with respect to the untreated control. Data are presented as the means ± SEM of three replicas.

## 5. Conclusions

In summary, we assembled here a theranostic platform made of graphene oxide and a tetra repeat sequence of HPRG.

Results of cell viability in human prostate cancer (PC-3), neuroblastoma (SH-SY5Y) and retinal endothelial (HREC) cells incubated with GO-immobilized peptide, both hybrids GO@T and mixtures GO+T, compared to the treatments with the free Tetra(HPRG)-Fam peptide, pointed to different binding capabilities of the HPRG backbone in the free and surface-immobilized conformational state.

In particular, the proliferative effect for the peptide alone and the anti-proliferative effect for the GO-immobilized peptide, detected both in PC-3 and HREC, were related to the predominance of poly-Proline II conformation of the peptide immobilized on the GO nanosheets. Also, results of confocal imaging analyses suggested the activation of different signaling pathways resulting by different peptide internalization and sub-cellular localization. The strong inhibitory effect observed on the migration of PC-3 and HREC cells in the presence of GO or GO@T hybrids was correlated to the PEG_2_ release.

Such findings may provide novel insights into the therapeutic potential of these nanocomposite. In fact, we demonstrated that a 21-mer peptide analogue mimics the anti-angiogenic activity of the whole HPRG protein and is also able to inhibit tumor growth when associated to the graphene oxide. Such a dual action could represent the basis for obtaining a new, more effective, anticancer therapeutic approach. To this aim, further studies are necessary to deepen the signaling pathway involved in the implementation of the overall function.

## Figures and Tables

**Table 1:** Prostaglandin (PGE_2_) production in PC-3 medium, after different treatments. Values (means ± SEM) are from three independent experiments (*n* = 3). ANOVA was used to compare PGE2 production in all experimental conditions. * *p* < 0.01 vs. control cells.

PC-3 Treatment	PGE_2_ (pg/mL)
CTRL	103.1 ± 9.2
GO (10 µg/mL)	64.5 ± 6.3 *
GO (12 µg/mL)	66.3 ± 6.0 *
GO (30 µg/mL)	60.2 ± 4.8 *
Tetra(HPRG)-Fam (2 µM)	78.1 ± 6.2 *
Tetra(HPRG)-Fam (4 µM)	71.7 ± 6.4 *
Tetra(HPRG)-Fam (6 µM)	76.3 ± 5.8 *
GO_A@T ([GO] = 10 µg/mL, [Tetra(HPRG)-Fam] = 2 µM	40.5 ± 3.7 *
GO_B@T ([GO] = 12 µg/mL, [Tetra(HPRG)-Fam] = 4 µM	44.3 ± 4.1 *
GO_A@T ([GO] = 30 µg/mL, [Tetra(HPRG)-Fam] = 6 µM	41.1 ± 4.8 *
GO_A+T ([GO] = 10 µg/mL, [Tetra(HPRG)-Fam] = 2 µM	62.2 ± 6.1 *
GO_B+T ([GO] = 12 µg/mL, [Tetra(HPRG)-Fam] = 4 µM	66.0 ± 5.5 *
GO_A+T ([GO] = 30 µg/mL, [Tetra(HPRG)-Fam] = 6 µM	57.8 ± 6.4 *

**Table 2:** PGE_2_ production in human retinal endothelial cells (HREC), after different treatments. Values (means ± SEM) are from three independent experiments (*n* = 3). Student’s *t*-test used to compare PGE_2_ production in all experimental conditions. * *p* < 0.01 vs. control cells.

HREC Treatment	PGE_2_ (pg/mL)
CTRL	71.9 ± 6.1
GO (10 µg/mL)	55.3 ± 4.2 *
GO (12 µg/mL)	50.9 ± 5.4 *
GO (30 µg/mL)	57.6 ± 5.2 *
Tetra(HPRG)-Fam (2 µM)	67.7 ± 5.8
Tetra(HPRG)-Fam (4 µM)	70.1 ± 5.5
Tetra(HPRG)-Fam (6 µM)	73.9 ± 6.3
GO_A@T ([GO] = 10 µg/mL, [Tetra(HPRG)-Fam] = 2 µM	25.4 ± 2.2 *
GO_B@T ([GO] = 12 µg/mL, [Tetra(HPRG)-Fam] = 4 µM	28.2 ± 3.8 *
GO_A@T ([GO] = 30 µg/mL, [Tetra(HPRG)-Fam] = 6 µM	23.5 ± 2.1 *
GO_A+T ([GO] = 10 µg/mL, [Tetra(HPRG)-Fam] = 2 µM	43.1 ± 5.2 *
GO_B+T ([GO] = 12 µg/mL, [Tetra(HPRG)-Fam] = 4 µM	46.4 ± 6.3 *
GO_A+T ([GO] = 30 µg/mL, [Tetra(HPRG)-Fam] = 6 µM	41.7 ± 4.8 *
